# A three-terminal non-volatile ferroelectric switch with an insulator–metal transition channel

**DOI:** 10.1038/s41598-021-03560-w

**Published:** 2022-02-09

**Authors:** Jaykumar Vaidya, R. S. Surya Kanthi, Shamiul Alam, Nazmul Amin, Ahmedullah Aziz, Nikhil Shukla

**Affiliations:** 1 Department of Electrical and Computer Engineering, University of Virginia, Charlottesville, VA 22904 USA; 2 Department of Electrical Engineering and Computer Science, University of Tennessee, Knoxville, TN 37996 USA

**Keywords:** Electrical and electronic engineering, Electronics, photonics and device physics

## Abstract

Ferroelectrics offer a promising material platform to realize energy-efficient non-volatile memory technology with the FeFET-based implementations being one of the most area-efficient ferroelectric memory architectures. However, the FeFET operation entails a fundamental trade-off between the read and the program operations. To overcome this trade-off, we propose in this work, a novel device concept, Mott-FeFET, that aims to replace the Silicon channel of the FeFET with VO_2_- a material that exhibits an electrically driven insulator–metal phase transition. The Mott-FeFET design, which demonstrates a (ferroelectric) polarization-dependent threshold voltage, enables the read current distinguishability (i.e., the ratio of current sensed when the Mott-FeFET is in state 1 and 0, respectively) to be independent of the program voltage. This enables the device to be programmed at low voltages without affecting the ability to sense/read the state of the device. Our work provides a pathway to realize low-voltage and energy-efficient non-volatile memory solutions.

The electric-field (E-field) induced non-volatile polarization switching in ferroelectrics makes them a promising candidate for developing non-volatile memory (NVM) technology. Conventionally, ferroelectric-based random-access memory (RAM) was realized using traditional ferroelectrics such as PZT, and showed energy-efficient operation, fast read as well as high endurance^1,2^. However, this ferroelectric memory technology was challenging to scale since ferroelectrics such as PZT typically exhibit a significant degradation in the ferroelectric response when the film thickness is scaled below 50 nm^3^. Consequently, the recent discovery of ferroelectricity in highly scaled HfO_2_- a material that is compatible with CMOS process technology- has generated immense interest in revisiting ferroelectric memory technology^4,5^. Particularly, the ability to integrate the ferroelectric directly into the gate of a field effect transistor (FET) has motivated active investigation of FeFET (Ferroelectric FET)-based non-volatile memory^6^. However, the FeFET design entails a fundamental trade-off between the programming and the read/sensing characteristics^7–9^. The objective of this work is to propose a pathway to overcome this trade-off and help reduce the programming voltage (at a fixed read current ratio) by replacing the Silicon channel by an alternate channel material, VO_2_ (vanadium dioxide), that exhibits the phenomenon of electrically driven insulator-to-metal transition (IMT).

A conventional FeFET involves a fundamental trade-off between the program voltage (write operation), and the MW (memory window) along with the corresponding read current distinguishability, expressed as I_bit_1_/I_bit_0_ (I_bit_1_ and I_bit_0_ are the sense currents measured corresponding to bit 1 and 0, respectively). Increasing the memory window and the corresponding I_bit_1_/I_bit_0_ requires the application of a significantly larger programming voltage. This is because in the FeFET configuration, the ferroelectric typically operates on a minor loop (not saturation loop) of the polarization versus voltage characteristics and improving the MW entails increasing the hysteresis by the application of a larger programming voltage. Moreover, these contending factors can become even more critical while operating the cell in a memory array where the parasitic currents from half-selected cells can further compromise the read distinguishability. Additionally, the larger program voltage also results in extremely large electric-fields (in excess of 10 MV/cm) across the interlayer (IL) between the ferroelectric and the Silicon channel which can adversely impact the reliability and the endurance of the device^8^. These trade-offs have been quantitatively analyzed in prior works^7,9^ including those by the authors^8,10^.

To illustrate the concept of the Mott-FeFET, we first develop models for the individual components of the device, namely, the ferroelectric HfO_2_ and the VO_2_ channel. Figure 1a shows the simulated polarization versus voltage characteristics of the ferroelectric HfO_2_ (*f*-HfO_2_) considered in this work. These characteristics have been simulated using the phenomenological Preisach’s model^11^ and have been calibrated to experimental data on 10 nm thick *f*-HfO_2_ films (in a metal/ferroelectric/metal capacitor configuration) reported by S. Mueller et al*.*^12^.

**Figure 1:**
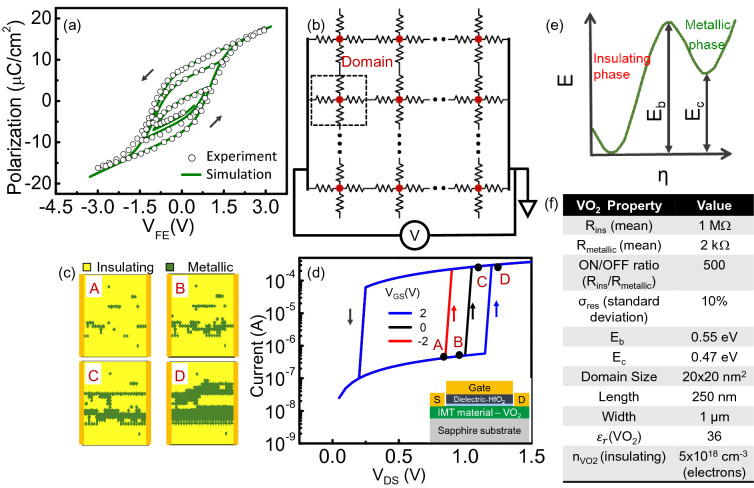
Modeling electrical response of the ferroelectric and VO_2_. (**a**) Polarization versus voltage characteristics of the ferroelectric HfO_2_ simulated using the Preisach’s model, and calibrated to the experimental data reported by Mueller et al.^12^ (**b**) Two-dimensional resistive network used to model the filamentary switching behavior across the IMT in VO_2_. (**c**) Evolution of the insulating and metallic phases across the electrically driven IMT in VO_2_ clearly showing the filamentary switching; the phases are indicated on the black IV curve in next panel. (**d**) Modulation of the threshold voltage for the IMT as a function of the applied electric field. Schematic of the device considered is also shown (inset) (**e**) Phase diagram of VO_2_ (**f**) Parameters used for simulation of VO_2_.

Next, we consider the electronically driven IMT in VO_2_. We note that while other oxides such as NbO_2_^13^, SmNiO_3_^14^ among others also demonstrate similar electronically driven IMT behavior, VO_2_ was our choice of IMT material in this work since it exhibits a large R_OFF_/R_ON_ ratio (> 10^4^)^15^, and more importantly, the modulation of the threshold voltage of the electrical IMT with gate electric field, critical to the Mott-FeFET operation, has been experimentally demonstrated in this material system^16,17^.

Two-terminal VO_2_ devices exhibit an electrically induced IMT that is characterized by an abrupt change in resistance at a particular applied voltage (V_T_: threshold voltage) as the device transitions from the insulating to the metallic state^18^. The transition is volatile, and the device returns back to the insulating state (metal-to-insulator transition; MIT) when the applied voltage subsequently drops below a threshold (V_H_: hold voltage), accompanied by hysteresis (V_T_-V_H_). To simulate the electronically induced IMT in VO_2_, we model the two-terminal VO_2_ device as a network of resistors that represent domains (Fig. 1b), as proposed in prior work^19^. Each resistance in this 2D network can undergo an IMT and MIT (metal to insulator transition) with a certain probability that is dependent on the voltage. Using the approach proposed by Madan et al*.*^15^ and Poklonski et al*.*^20^, we model the switching probability for a domain using the following equations:

1 $$P_{IMT} = e^{{\frac{{ - \left( {E_{b} - \frac{q\Delta V}{\gamma }{ }} \right)}}{kT}}}$$

And,

2 $${\text{P}}_{{{\text{MIT}}}} = {\text{e}}^{{ - \frac{{\left( {{\text{E}}_{{\text{b}}} - {\text{E}}_{{\text{c}}} } \right)}}{{{\text{kT}}}}}}$$

where P_MIT_ and P_IMT_ are the probabilities of a domain undergoing MIT and IMT, respectively. E_b_ and E_c_ are energy barriers as defined in Fig. 1e, and γ is a geometric factor^15^.﻿

It can be observed that applying a voltage increases the probability of a domain undergoing IMT. Additionally, we also consider a gaussian distribution for the resistance values representing a domain to account for the heterogeneity in the film. The parameters for the IMT in VO_2_ are shown in the table in Fig. 1f. A detailed discussion of the electronic IMT in VO_2_ has been included in supplement S3.

The voltage-induced IMT in the device can be explained as follows. Initially, all the domains are in the insulating state (at zero bias). As the voltage across the device is increased, a few domains (probabilistically) undergo IMT, serving as the nucleation centers for the metallic phase. As the domain transitions to the metallic state, the voltage drop across the domain reduces, leading to a corresponding increase in the voltage drop across other domains, which in turn, increases their probability of switching. This process generates an avalanching effect that eventually creates a metallic filamentary bridge between the electrodes, resulting in an abrupt change in resistance of the device; the width of the filament depends on the current passing through the device- an effect that is captured by our model as well (Fig. 1c). Furthermore, the presence of filamentary conduction has been experimentally shown in prior work^21^. Similar (albeit weaker) avalanching behavior is observed during MIT leading to an abrupt increase in resistance as the device turns OFF. We also note that the switching in VO_2_ is stochastic (see supplement S1a) which consequently has important implications for the design of the memory array.

Additionally, in the three-terminal device configuration with a gate dielectric, Kim et al*.*^16^, and Tabib-Azar et al.^17^ experimentally demonstrated that the threshold voltage of the VO_2_ channel can be modulated by applying an electric field across the gate—a property crucial to the Mott-FeFET operation. We note that the gate-field alone does not induce the IMT but aids the transition. We model this behavior *phenomenologically* by modifying Eq. (1) to include the effect of surface potential induced by the gate: $P_{IMT} = e^{{\frac{{ - \left( {E_{b} - \frac{q\Delta V}{\gamma } - \alpha q\psi_{s} } \right)}}{kT}}}$. Here, Ψs is the VO_2_ surface potential due to the gate and α is the coupling constant introduced between the gate-induced surface potential and the IMT transition in the VO_2_ device (set to 0.5). Since the gate-field alone does not induce the transition but modifies the threshold voltage (V_T_), we model this effect as the surface potential modulating the probability of the domain switching, which subsequently, manifests as the change in the (drain-to-source) V_T_ required to induce the IMT, as shown in Fig. 1d. Additionally, we do not consider this effect in the MIT characteristics since the operation of our proposed device as a memory cell does not rely on the MIT, as well as due to the absence of experimental data. We also emphasize that while the proposed model can explain the experimental behavior shown by Kim et al*.*^16^ and Tabib-Azar et al.^17^*,* it is important to qualify that model is phenomenological in nature; the exact physics of the electrically induced Mott-Peierls IMT in VO_2_ still remains an active, ongoing investigation.

Another important aspect of the gate electric-field induced modulation is that while it has a significant influence on the threshold voltage for the IMT (by influencing the nucleation dynamics of the metallic phase), its impact on the metallic and insulating states is minimal. This because the high conductance state of the VO_2_ is essentially metallic in nature which limits the penetration of the gate field. Furthermore, the impact of the electric field on the resistivity of insulating state is also expected to be minimal^22^. This can be attributed to the formation of small polarons that result from the gate-induced charge coupling to the lattice, as shown in our prior work^23^, as well as in other works^24^. These polarons screen the electric field, and subsequently, limit its penetration to a few (1–2) monolayers, resulting in minimal effect on the conductivity. This ensures that for a current sensing-based reading scheme, the read distinguishability i.e., I_bit_1_/I_bit_0_ would essentially be constant, irrespective of the programming voltage / field applied at the gate. This behavior is fundamentally different from that of a conventional Si transistor where the gate field strongly controls the channel resistance, and consequently, the channel current.

The Mott-FeFET design (Fig. 2a) aims to integrate the non-volatile polarization switching in the ferroelectric gate with the abrupt resistance switching across the electrically driven IMT in VO_2_ channel. The expected operation of the Mott-FeFET can be described as follows: the polarization state (up or down) of the ferroelectric gate is used to represent the information bit to be stored and can be programmed (write operation) by applying the program voltage across the gate of the device (details of the polarity are discussed in the array operation). The resulting surface potential associated with the (different) polarization states of the ferroelectric modulates the threshold voltage (V_T,1_, V_T,0_) of the IMT in the VO_2_ channel i.e., one polarization state results in a larger threshold voltage than the other state, creating a memory window (ΔV_T_ = V_T,0_ − V_T,1_) as shown in Fig. 2b. Subsequently, the state of the memory can be sensed by applying an appropriate read voltage V_READ_ such that V_T,1_ < V_READ_ < V_T,0_. This ensures that if the memory cell is in state 1, a large drain current, corresponding to the metallic state of VO_2_, will be sensed whereas state 0 will produce a significantly smaller drain current owing to the insulating nature of the channel. Therefore, the VO_2_ channel can be considered as a ‘selector’ whose threshold voltage depends on the state of the memory (i.e., ferroelectric gate).

**Figure 2:**
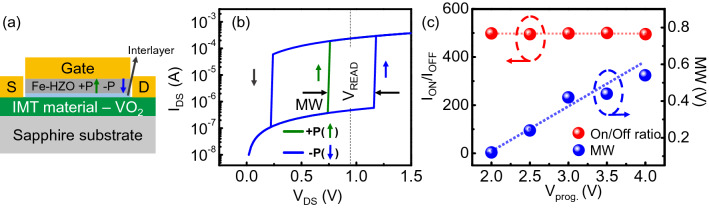
Mott-FeFET operation. (**a**) Schematic of the proposed Mott-FeFET. (**b**) I_ds_ versus V_ds_ characteristics of the VO_2_ channel as a function of the ferroelectric polarization. (**c**) Observed ratio between read currents corresponding to state 1 and 0, as a function of the applied program voltage. It can be observed that I_ON_/I_OFF_ ratio, unlike in a conventional FeFET remains constant.

We explore the operation of the proposed Mott-FeFET by integrating the models developed above for the individual components, namely, polarization switching in the ferroelectric and the electronically induced abrupt resistance switching in VO_2_. Moreover, the ferroelectric polarization interacts with the VO_2_ channel through the surface potential which is calculated by modeling the capacitance response of the gate stack. The surface potential, which depends on the state of polarization of the ferroelectric, subsequently, modulates the probability of the switching (from insulating to metallic state) in the VO_2_ domains, resulting in a ferroelectric polarization state dependent IMT threshold voltage. A finite interlayer at the interface between the ferroelectric and the VO_2_ is also considered. Using this framework, we simulate the characteristics of the Mott-FeFET, as shown in Fig. 2b. It can be observed that the threshold voltage for the IMT in the VO_2_ channel varies by ~ 0.5 V, opening a memory window that can facilitate its use as a non-volatile storage element.

We note that while the memory window (i.e., ΔV_T_ = V_T,0_ − V_T,1_) is sensitive to the polarization, which in turn depends on the voltage used to program the ferroelectric, the I_bit_1_/I_bit_0_ ratio (within the memory window) is almost insensitive to the program voltage of the ferroelectric, as observed in Fig. 2c. This is because the current distinguishability is primarily decided by the R_OFF_ and R_ON_ of the VO_2_ which are relatively insensitive to the gate-field, as discussed above. The insensitivity of the I_bit_1_/I_bit_0_ ratio to the program voltage can facilitate scaling of the program voltage without adversely impacting the read/sense characteristics and margins.

Next, we evaluate the operation of the Mott-FeFET as a memory element in a non-volatile memory array. We consider the NOR memory architecture as shown in Fig. 3a, where the basic building block of the array consists of the Mott-FeFET as the memory element whose gate is connected to a simple MOSFET, which functions as the access device. This architecture, which consists of a separate word-line to read (WLR) from, and write (WLW) to, a cell is similar to that proposed for FeFET-based memory arrays^25^. A phenomenological Verilog-A model is used to simulate the Mott-FeFET whereas the DGXFET NMOS model, available in the IBM 65 nm CMOS 10LPe process, is used for the transistor.

**Figure 3:**
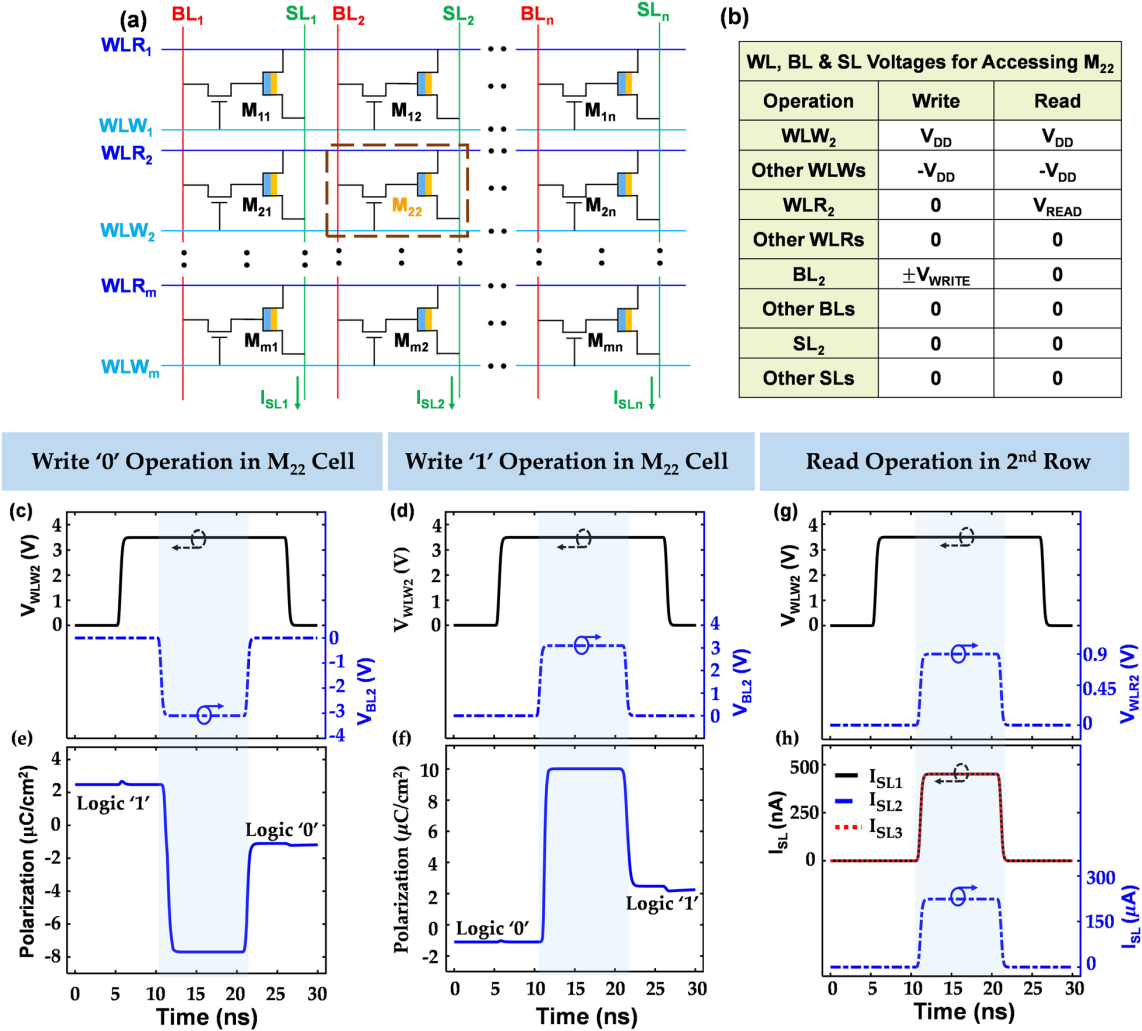
Mott-FeFET array operation. (**a**) Schematic of the proposed Mott-FeFET-based memory array. (**b**) Biasing scheme for WLWs, WLRs, BLs and SLs to access a memory cell (M_22_ here). The results presented here are for a 3 × 3 array. Time dynamics of the bias voltages applied across WLW2 and BL2 during (**c**) write ‘0’ and (**d**) write ‘1’ operations in the M_22_ cell. During write operation, the other WLWs and BLs are biased at V_DD_ and 0 V, respectively, and all the WLRs and SLs are biased at 0 V. Time evolution of the ferroelectric polarization for (**e**) write ‘1’ → ‘0’ and (**f**) write ‘0’ → ‘1’ operations in the M_22_ cell. (**g**) Temporal dynamics of the bias voltages of WLW2 and WLR2 for read operation of the cells in the second row. The bias voltages for other WLWs, WLRs, BLs and SLs are kept constant at specific levels (shown in (**b**)). (**h**) SL currents during read operation. This array architecture facilitates reading the entire row in one cycle. Here, we only read the second row. M_21_ and M_23_ cells were initialized with logic ‘0’ and M_22_ with logic ‘1’ before read operation. The effect of the stored memory state is observed in the corresponding SL currents. SL_1_ and SL_3_ currents are 450 nA due to logic ‘0’ in M_21_ and M_23_ and SL_2_ current is 225 µA due to logic ‘1’ stored in M_22_.

The biasing scheme for reading from-, and writing to-, a particular cell of the memory array is designed to facilitate successful reading and writing operations, without disturbing other cells in the array. Here, we consider the illustrative example of accessing the M_22_ cell in a 3 × 3 array. The corresponding biasing conditions are shown in Fig. 3b. Figure 3c,d show the bias voltages applied to WLW_2_ and BL_2_ (connected with M_22_) for the write ‘0’ and write ‘1’ operations, respectively. The bias conditions for the other WLWs, BLs, WLRs, and SLs are in accordance with those listed in the table in Fig. 3b. The WLW_2_ is asserted to turn ON the access transistors of the second row, and a suitable programming voltage ($\pm$ *V*_WRITE_) is applied to the BL_2_ with the objective to facilitate sufficient bias at the gate of the ferroelectric to modulate the polarization, as needed. Figure 3e,f show the evolution of the ferroelectric polarization during write ‘1’ → ‘0’ and write ‘0’ → ‘1’ operations, respectively. The appropriate choice of bias conditions eliminates the possibility of the accidental write into the other cells of the array (details discussed in supplement S4).

The proposed array architecture also facilitates reading all the cells in a row in one cycle. To illustrate this, we initially store ‘0’ in M_21_ and M_23_ and ‘1’ in M_22_ cells belonging to the second row of the 3 × 3 array. To read from a cell, we utilize the *I*_*DS*_ − *V*_*DS*_ characteristics of Mott-FeFET (at zero gate bias) shown in Fig. 2b. Figure 3g shows the bias conditions for the WLW_2_ and WLR_2_ and the corresponding SL currents are shown in Fig. 3h. It can be observed that the SL connected to the cell with logic ‘0’ generates ~ 450 nA whereas the logic 1 produces a current of ~ 225 µA on SL. This difference in the SL current is used for the sensing of the stored memory states using current sense amplifiers^26,27^ (see supplement S4 for more details on the sensing mechanism).

## Discussion

The goal of this work is to propose and elucidate a new device concept, Mott-FeFET, that aims to overcome the read–write trade-offs in conventional Silicon FeFET designs by leveraging the unique properties of IMT. It showcases an example of how novel functional materials and their properties (here, the IMT in VO_2_) can be used to overcome the design challenges of Silicon devices. While the focus of the work is primarily to describe the operational characteristics and functional properties of the Mott-FeFET, it is important to note that the physical realization of such a device would inevitably need to address important challenges such as the integration of the *f*-HfO_2_ on VO_2_ while retaining their functional properties, the role of the interfacial layer and interface states among others; overcoming these concerns will be critical to the eventual practicality of such a device. Additionally, we also note that the underlying physics of the electrically induced IMT in VO_2_ as well as how an external electric field affects the IMT still remains to be fully understood. However, the present work helps nucleate the new device concept, and motivates the investigation of the above questions, which can subsequently, enable energy-efficient and high performance non-volatile random-access memory.

## Methods

The Mott-FeFET was simulated using the Xyce Parallel Electronic Simulator^28^-a SPICE-compatible circuit simulator provided by Sandia National Laboratory, interfaced with MATLAB; the 2D resistive network was implemented in Xyce whereas the domain switching was analyzed in MATLAB. The experimentally calibrated ferroelectric characteristics were simulated using the Preisach model implemented in MATLAB. For array-level simulations, a phenomenological compact model was utilized for the individual memory elements. This compact model was implemented in Verilog-A and was calibrated with the polarization dynamics and electrical characteristics obtained from the physics-based predictive model for Mott-FeFET. The DGXFET NMOS model from the IBM 65 nm CMOS 10LPe process was utilized to simulate the access transistors in the memory array.

## Supplementary Information

Supplementary Information.
