# Supplementary material for: A three-terminal non-volatile ferroelectric switch with an insulator–metal transition channel
Source: Sci Rep. 2022 Feb 9;12:2199. doi: 10.1038/s41598-021-03560-w (PMC8828903; doi:10.1038/s41598-021-03560-w)
Supplement: Supplementary file 1 — Supplementary Information. [file 41598_2021_3560_MOESM1_ESM.pdf]

Supplementary Information

A Three-terminal Non-Volatile Ferroelectric Switch with an Insulator-Metal Transition Channel

Jaykumar Vaidya[1,a)], R. S. Surya Kanthi[1,a)], Shamiul Alam[2], Nazmul Amin[2], Ahmedullah Aziz[2], Nikhil Shukla[1]*

[1]Department of Electrical and Computer Engineering, University of Virginia, Charlottesville, VA- 22904, USA

[2]Department of Electrical Engineering and Computer Science, University of Tennessee, Knoxville, TN 37996, USA

a) equal contribution

*e-mail: ns6pf@virginia.edu

S1. Stochastic switching in VO_2

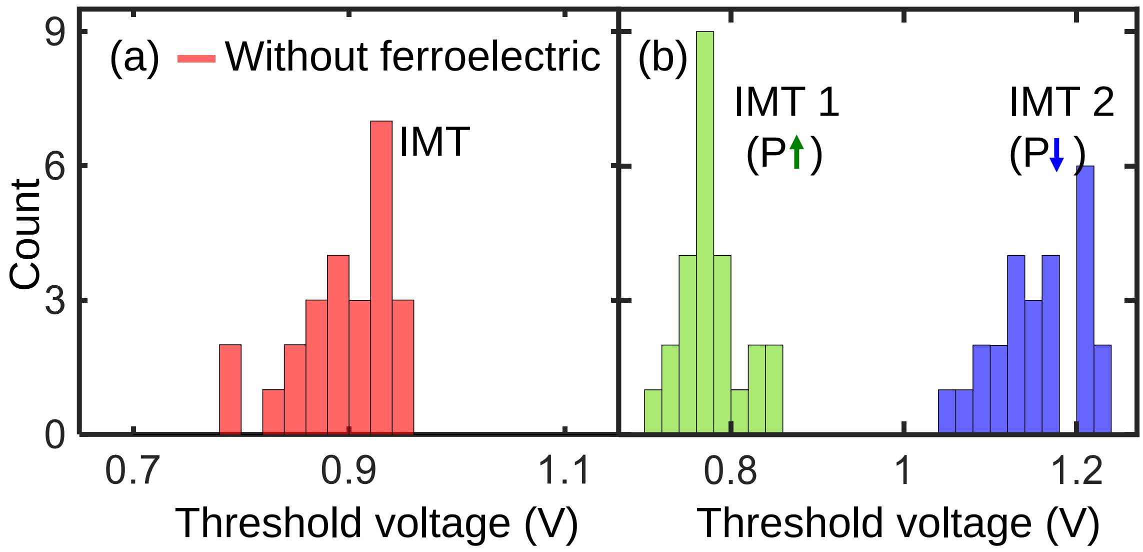

Fig. S1. Stochastic nature of switching in VO_2. (a) Distribution of IMT threshold voltage in 2 terminal VO_2 device (25 sweeps were considered). (b) Distribution of threshold voltages corresponding to the two states of the ferroelectric in the Mott-FeFET (25 sweeps were simulated).

S2. Electrically driven IMT in VO_2 (Experimental characteristics)

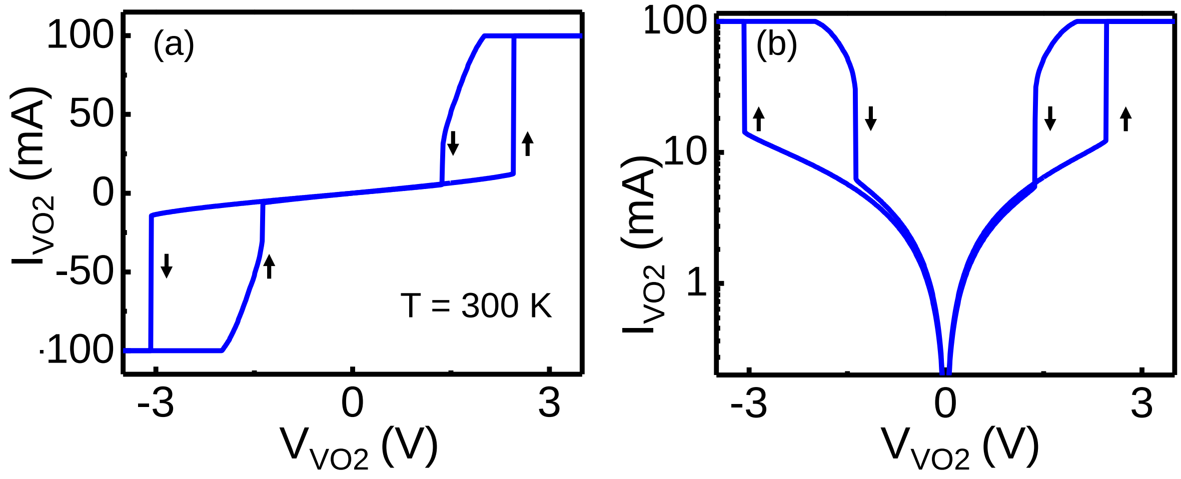

Fig. S2. Current vs. voltage characteristics of VO_2. Illustrative current vs. voltage characteristics of VO_2 measured experimentally in a two-terminal device configuration shown using a (a) Linear scale, (b) Logarithmic scale for the current axis.

Illustrative I-V characteristics showing electrically induced IMT and MIT in VO_2 marked by an abrupt and hysteretic change in resistance. The device length and width was 10 µm.

S3. Nature of the electrical IMT in VO_2

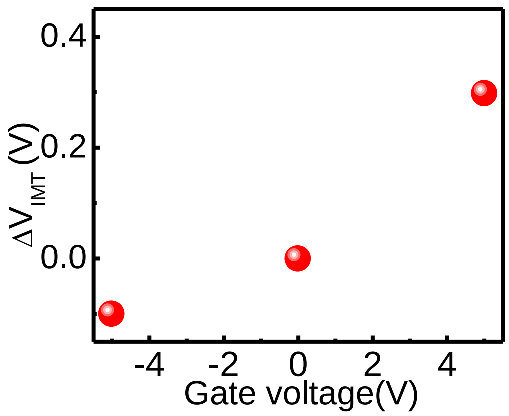

Fig. S3 Variation in IMT threshold voltage as a function of the applied gate voltage as reported by Tabib-Azar *et al* [3].

The origin of the IMT in VO_2 has been the subject of intense research and debate[1]. Various models based on varying levels of contribution from a Mott-Hubbard type transition and a Peierls-like structural instability have been proposed to explain the transition. However, a comprehensive understanding of the exact origins of the IMT in VO_2 still remains elusive. Consequently, this also implies that the exact mechanism of how an external stimulus such as an electric field affects the IMT in VO_2 also remains to be completely understood.

Electronically driven IMT in VO_2: Two-terminal VO_2 devices exhibit an IMT when a voltage is applied across the VO_2 channel. In this configuration, both electric-field and current-induced Joule heating effects are present[2]. While the exact origin of this transition is also disputed, there is increasing evidence of the preponderance of electro-thermal effects[3,4]. In a three-terminal device, a true gate-field induced IMT has not been demonstrated, although several useful features of the interaction between the (gate) electric-field and the VO_2 channel have been revealed (we note that Nakano *et al*[5]. demonstrated a non-volatile phase transition using ionic liquid gating where the role of ionic diffusion and the electric field are challenging to deconvolute). A key feature of the application of an electric field (through the gate) on the VO_2 channel is that it modulates the (threshold) voltage required at the source-drain to induce IMT[6] - a property that facilitates the design of the Mott FeFET proposed here. One possible explanation for this behavior is that even though the magnitude of the electric-field required to induce an IMT is significantly larger than that which can supported by a solid state dielectric, the field modulates the nucleation probability of the metallic phase (field induced nucleation) resulting in the change in the threshold voltage. We therefore propose a phenomenological model to emulate this behavior.

S4. Design and operation of Mott-FeFET array

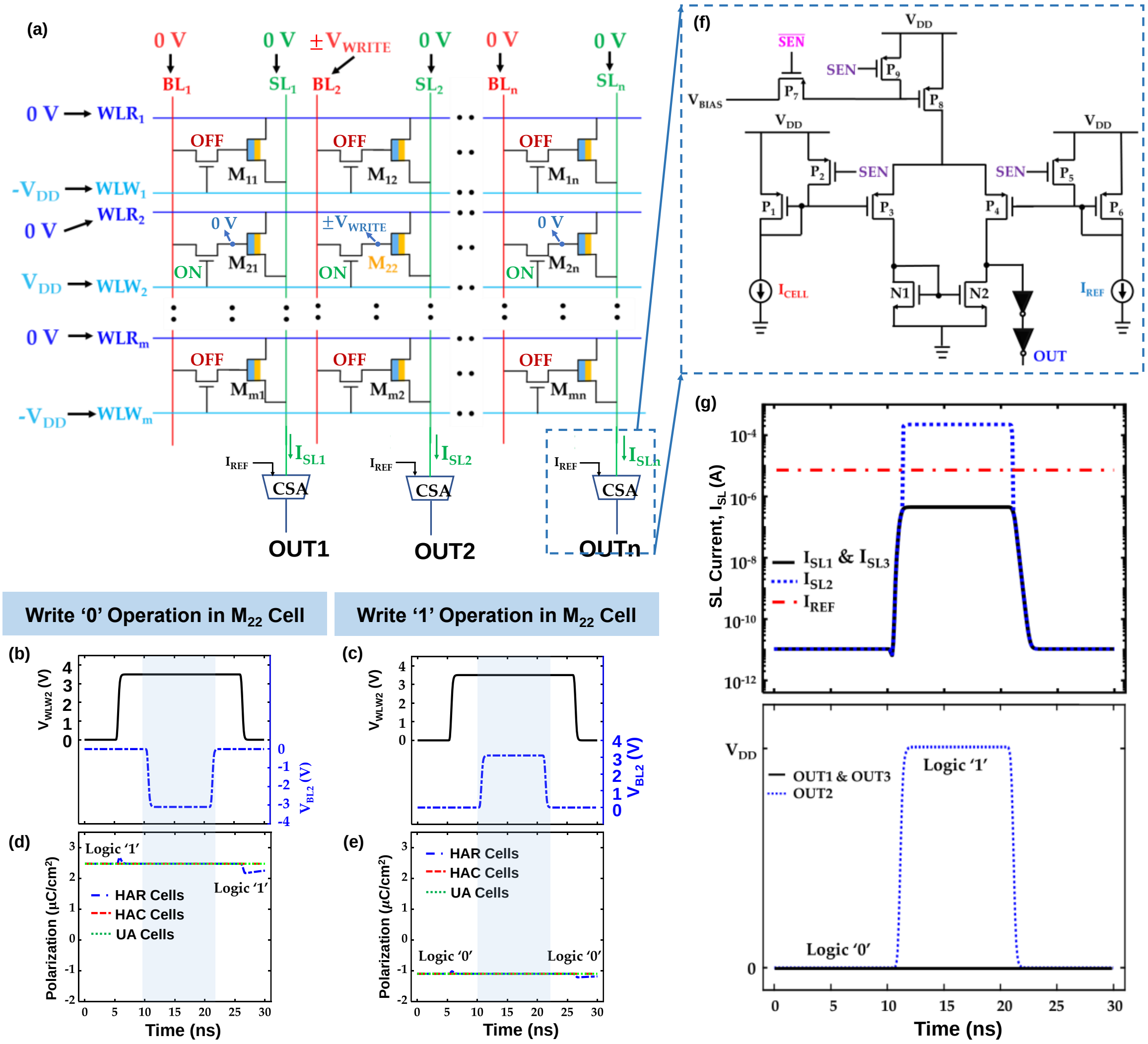

Fig. S4: Additional Details for the Write Operation and Sensing Mechanism of the Array. (a) Biasing conditions for WLWs, WLRs, BLs, and SLs during the write operation in M_{22} cell. The choice of biasing for WLWs and SLs ensures that only the access transistors in the same row with the accessed cell turn on while all the other transistors remain off. Now, the choice of biasing for BLs ensures that only the accessed cell (M_{22}) gets the write voltage ($\pm V_{WRITE}$) at the gate while all the other cells of that row get 0 V at their gate terminals. A current sense amplifier (CSA) is connected to each SL for the sensing purpose. (b) & (c) Time dynamics of the bias voltages of WLW_2 and BL_2 during write '0' and write '1' operations in M_{22}, respectively. (d) & (e) Preservation of the memory states of HAR, HAC, and UA cells during write '0' and write '1' operations, respectively due to the suitable choice of biasing for the WLWs and BLs. (f) The circuit schematic of the current sense amplifier. (g) SL currents and CSA outputs for read operation in the second row.

Figure 3 of the main text demonstrates the write and read operations in a memory cell within a 3 × 3 array. Additional details are described here. During write/read operations, it is important to ensure the following:

(i) The stored data in the inactive cells does not get disturbed during the read/write operations

(ii) During the read operation, the SL current shows distinguishable difference for low/high memory states

We first discuss the possibility of the accidental manipulation of the data stored in the inactive cells. Note, in the Mott-FeFET based memory cells, the write and the read operations are performed using the gate and drain bias, respectively. For write (read) operation, suitable V_{WRITE} (V_{READ}) is applied as V_{GS} (V_{DS}), while V_{DS} (V_{GS}) is kept at 0 V. Thus, accidental programming in the inactive cells can be avoided while reading from the active cells. Now, during the write operation, the biasing conditions for the WLWs, WLRs, BLs and SLs (shown in Fig. S4a) are carefully chosen to ensure that, only the accessed cell gets the programming voltage at the gate terminal. The biasing of WLWs (Fig. S4b,c) ensures that the access transistors of half-accessed column (HAC) and unaccessed (UA) cells remain off[7]. Although the access transistors of half-accessed row (HAR) cells turn ON, the BL biasing (Fig. S4b,c) ensures that the HAR cells get 0 V at their gate terminals. Therefore, the memory states of the HAR, HAC, UA cells remain undisturbed during both the write '0' (Fig. S4d), and the write '1' (Fig. S4e) operations.

Next, we discuss the distinguishability in the SL currents during the read operation. We utilize the difference in the SL currents to sense the memory states stored in the cells of an array. Fig. 4h in the main text shows the SL currents for the read operation of the memory cells in the second row. It clearly shows that the SL currents provide sufficient distinguishability between logic '0' and logic '1' states. For sensing, a current sense amplifier (CSA) is connected to each SL, as shown in Fig. S4a. Figure S4b shows the schematic of the CSA[8] that we have used in this work. The reference current (I_{REF}= 10 μA) is appropriately chosen to obtain different binary outputs (0 and V_{DD}) for logic '0' and logic '1', respectively. Figure S4c shows the SL currents and corresponding logic outputs of the CSA during the read operation.

References

1. Shao, Z., Cao, X., Luo, H. & Jin, P. Recent progress in the phase-transition mechanism and modulation of vanadium dioxide materials. *NPG Asia Mater.* **10**, 581–605 (2018).

2. Farjadian, M. & Shalchian, M. Hybrid Electrothermal Model for Insulator-to- Metal Transition in VO_2 Thin Films. *IEEE Trans. Electron Devices* **68**, 704–712 (2021).

3. Tabib-Azar, M. & Likhite, R. Nano-Particle VO_2 Insulator-Metal Transition Field-Effect Switch with 42 mV/decade Sub-Threshold Slope. *Electronics* **8**, 151 (2019).

4. P, A., Chauhan, Y. S. & Verma, A. Vanadium dioxide thin films synthesized using low thermal budget atmospheric oxidation. *Thin Solid Films* **706**, 138003 (2020).

5. Nakano, M. *et al.* Collective bulk carrier delocalization driven by electrostatic surface charge accumulation. *Nature* **487**, 459–462 (2012).

6. Kim, H.-T. *et al.* Mechanism and observation of Mott transition in VO_2 -based two- and three-terminal devices. *New J. Phys.* **6**, 52–52 (2004).

7. Aziz, A., Jao, N., Datta, S., Narayanan, V. & Gupta, S. K. A computationally efficient compact model for leakage in cross-point array. in *2017 International Conference on Simulation of Semiconductor Processes and Devices (SISPAD)* 141–144 (IEEE, 2017). doi:10.23919/SISPAD.2017.8085284.

8. Chang, M.-F. *et al.* An offset-tolerant current-sampling-based sense amplifier for Sub-100nA-cell-current nonvolatile memory. in *2011 IEEE International Solid-State Circuits Conference* 206–208 (IEEE, 2011). doi:10.1109/ISSCC.2011.5746284.
